# Strategic recruitment and retention for pediatric research: a systematic review and meta-analysis

**DOI:** 10.3389/fped.2026.1786388

**Published:** 2026-04-10

**Authors:** Sergio Monares, Madeleine Godfrey, Ava Heinegg, Faith Taliaferro, Peter P. Moschovis, Wanda I. Gonzalez, Lael M. Yonker

**Affiliations:** 1 Mucosal Immunology and Biology Research Center, Massachusetts General Hospital, Boston, United States; 2 Department of Pediatrics, Massachusetts General Hospital, Boston, United States; 3 Harvard Medical School, Boston, MA, United States; 4 Adolescent and Pediatric Medicine, Massachusetts General Hospital Chelsea Healthcare Center, Chelsea, MA, United States; 5 Department of Pediatrics, University of Texas Southwestern Medical Center, Dallas, United States

**Keywords:** participant engagement, pediatric research, recruitment strategies, retention strategies, study design

## Abstract

**Objective:**

To evaluate the effectiveness of recruitment and retention strategies in pediatric clinical studies, focusing on achieving adequate representation across a range of demographic considerations.

**Study design:**

A systematic review was conducted using PubMed, PsycNET, and Cochrane databases to identify articles published between January 1, 1993, and September 1, 2024. Inclusion criteria targeted general pediatric studies emphasizing recruitment diversity. We compared success rates of these strategies across different races and ethnicities, targeted age groups, and community location, to current published national recruitment rates in pediatric research.

**Results:**

Of the 2,272 studies identified, 23 studies were included that detailed specific recruitment and retention strategies specifically targeting diverse pediatric populations for general pediatric health topics. These studies enrolled a total of 5,482 participants with ages ranging from 0.55–19.8 years of age for child and adolescent populations, and a mean parental age of 35.5 years for studies targeting parents of children. Community-based recruitment strategies were most effective for engaging underrepresented populations. Family services and flexible scheduling were particularly effective for parents and young children, while monetary compensation and group-oriented efforts resonated more with adolescents. Retention strategies, including flexible scheduling, family services, and compensation, were successful across populations.

**Conclusion:**

Tailored recruitment and retention strategies addressing cultural, social, and logistical needs are essential for ensuring diversity of age, race, ethnicity and geographic location in pediatric research. Community-based strategies enhanced recruitment, while compensation and logistical incentives, such as follow-up reminders and family services improved retention. Addressing data gaps on recruitment and retention efforts are critical for future research to achieve adequate representation and improve health outcomes in pediatrics.

## Introduction

Inclusion of children in clinical trials is essential for advancing care for pediatric populations (1). As with adults, representation must reflect the targeted pediatric population to order to achieve generalizability of scientific findings across diverse pediatric populations (2). Imbalances in pediatric clinical trial participation may translate into healthcare inequities (3), unintended biases, and inaccurate assumptions broadly affecting population health and limiting medical advances (4). Reviews in adult populations have underscored the importance of culturally tailored recruitment strategies, trust-building interventions and practical accommodations to mitigate barriers to participation (5–7). However, the applicability of these findings to pediatric research remains unclear due to the unique ethical, social and logistical considerations inherent in studies involving children.

Limitations to participation in pediatric research include complex historical and social barriers (8–10). These barriers include historical mistrust in research institutions, structural racism, prior unethical research practices, and ongoing disparities in access to healthcare and research infrastructure (5, 8, 11). In pediatric research specifically, these concerns may be amplified by parental fear of data misuse and perceived risks to children (6, 12). Recognizing these structural and relational factors is a requirement to understand why tailored recruitment approaches are necessary.

While recent reviews have addressed recruitment barriers in specific contexts, such as disease-specific or genomics trials (13–15), few have provided a comprehensive analysis of strategies aimed at increasing recruitment of understudied populations across general pediatric health research. This gap underscores the need to synthesize evidence on recruitment and retention strategies broadly targeting children for clinical research.

Practical barriers have also hindered pediatric inclusion in research, particularly when participation depends on parents and guardians who may need to balance multiple responsibilities within the family unit. These practical barriers, such as transportation difficulties, childcare needs, and scheduling conflicts, along with overly complex consent forms, unintentionally create barriers to participation (2, 16). These additional considerations compound existing hesitancy towards pediatric research, exacerbating underlying inequities in research participation.

To address recruitment and retention limitation in pediatric clinical studies, we conducted a systematic review evaluating the strategies associated with higher enrollment in recruitment and retention for pediatric subjects across diverse populations. Unlike prior reviews that focus on specific diseases or recruitment strategies, our systematic review uniquely evaluates the effectiveness of recruitment and retention practices across a broad spectrum of general pediatric healthcare studies, prioritizing enrollment of underrepresented ethnic groups.

The focus on general pediatrics rather than specific pediatric disease populations was chosen to avoid biases associated with recruitment through subspecialty clinics. For example, recruiting cystic fibrosis patients through a Cystic Fibrosis Center or recruiting pediatric cancer patients through a Cancer Center require different recruitment and retention tactics, as these individuals have an established, trusting relationship with the clinical and research teams. Additionally, research is often integrated into clinical care and research efforts likely directly impact their daily lives or medical outcomes. These kinds of recruitment and retention tactics (while valuable for specific research) limit generalizability to broader pediatric populations and obscure efforts to tailor recruitment for specific individuals and communities. Thus, we investigated generalizable recruitment and retention efforts based on racial/ethnic background, targeted age group (children, adolescents, parents), and by community (urban/rural) location. This focused systematic review contributes a novel synthesis of evidence on practices promoting comprehensive representation in pediatric research, addressing gaps in the literature and providing actionable insights for future studies. In order to achieve this, the review was guided by the following structured questions: (1) Which recruitment strategies are associated with improved enrollment of underrepresented pediatric populations in research? (2) Do recruitment outcomes vary according to age group, race/ethnicity, or community type? and (3) Which retention strategies are associated with sustained participation over time? These questions informed the search strategy, eligibility criteria, and analytic framework presented here.

## Methods

### Selection criteria

We performed a systematic review with the goal of identifying research articles specifically focused on attaining enrollment of underrepresented populations through engagements on topics applicable to general pediatrics. The articles chosen for the systematic review had to be published between January 1, 1993—January 1, 2024, due to the National Institutes of Health (NIH) Revitalization Act of 1993. This act mandated that NIH-funded clinical studies include women and minorities in their study populations (17), resulting in an increase in minority-based recruitment as well as more studies reporting their demographic breakdowns. The papers selected required: a human subject population focusing on pediatric subjects to examine which strategies effectively target children and their guardians; a generalizable study topic to avoid skewing subject demographics and studies that target a highly motivated or small patient subset and some form of subject interaction, such as a survey or study drug. A “generalizable study topic” was defined as a research focus applicable to broadly encountered pediatric health concerns in community or primary care settings (obesity, asthma, preventive care, health behaviors), rather than highly specialized tertiary care conditions or rare diseases that may involve uniquely motivated or narrowly defined patient populations. Our objective was to include studies addressing broad pediatric health topics relevant to general pediatrics while examining recruitment and retention strategies across diverse demographic groups. Accordingly, we focused our search on publications that reported demographic considerations such as race, ethnicity, age, and community location in study recruitment.

The databases used for locating relevant publications included PubMed, Cochrane Review, and PsycNet. The search terms were the following keywords in combination: “pediatrics” or “adolescence” and “research inclusion” and “diversity”. Search terms were selected to capture broadly applicable pediatric research focused on inclusion and diversity while avoiding restriction to specific disease areas. We prioritized general terminology to maximize identification of studies conducted in general pediatric settings.

This initial search yielded 2,272 articles which were then filtered to exclude articles published before 1993 and articles that were protocols, meta-analysis or reviews (*n* = 1,913). This left 357 articles to be assessed for eligibility. Detailed exclusion criteria applied during full-text screening are summarized in Supplementary Figure S1 (PRISMA flow diagram).

Two researchers conducted the initial screening of articles. To ensure a comprehensive search, the reference lists of selected papers were also reviewed by a third researcher to identify additional studies focusing on minority populations. Of the 357 articles, 334 articles were excluded for not including human subjects, being unrelated to demographic considerations, irrelevant to general pediatrics, not including recruitment strategies, or for being descriptive without reporting results. Twenty-three studies were included based on the selection criteria previously described (18–39) Supplementary Figure S1 presents the PRISMA flow diagram illustrating the study identification, screening, and selection process according to the established inclusion and exclusion criteria.

### Data selection process

The following study characteristics were extracted from each publication, as available: medical topic or disease studied; study type; number of subjects enrolled; demographic characteristics of recruited and retained participants (including age, sex, gender, sexual orientation, race, and ethnicity); geographic location of the study; classification of community type (urban, suburban, or rural) and detailed descriptions of recruitment and retention strategies.

Community type was categorized based on definitions provided within each publication. When not explicitly stated, classification was inferred from the described study setting (central hospital systems were categorized as urban, whereas school districts or community centers serving non metropolitan areas were categorized as suburban or rural based on contextual description). Extracted information was reviewed independently by at least two of the three researchers to verify accuracy.

### Statistical analysis

Descriptive statistics were employed to characterize the studies. The number of studies using each recruitment and retention strategy was quantified. To calculate recruitment percentages, we divided the number of subjects recruited by the total number offered participation, and to calculate retention percentages, we divided the number retained by the total recruited, each expressed as a percentage. Forest plots allowed visualization of recruitment and retention strategy efficacy for different underrepresented populations (median percentage and 95% confidence intervals of the specified population recruited/retained), as well as for different locations and age groups. Racial and ethnic enrollment data was compared against reported national averages (40).

## Results

The findings are presented in alignment with the guiding review questions, first examining recruitment strategies associated with higher enrollment, followed by stratified analyses by age, race/ethnicity and community type and concluding with retention strategies. We identified 23 publications that described general pediatric clinical studies emphasizing recruitment of underrepresented populations in their study design. Of these 23 publications, 13 (57%) were observational/survey studies, whereas 10 (43%) involved an intervention. Study topics were varied, including topics such as pediatric obesity and nutrition, healthcare communications and quality strategies, and asthma interventions. Studies sought to enroll children across a range of ages, with several studies spanning multiple age targets. The majority (*n* = 13, 57%) of studies targeted urban populations, whereas 7 studies targeted suburban, and 7 studies targeted rural communities, across various international and national regions. All 23 studies were funded by federal sources, and 8 had additional non-federal funding. Of note, some of these studies employed multiple modalities, targeted multiple locations or populations, and received multiple sources of funding. Table 1 outlines the general characteristics of the studies included in the systematic review.

**Table 1:** Study characteristics. Study type, enriched demographic group, topic studied, target age population, community type of participants, region where study was performed, and reported funding sourced were listed for studies included in this systematic review.

Study characteristics (*N* = 23)	*n*	%
Study type		
Observational	13	57
Interventional	10	43
Enriched Demographic Group		
Hispanic/Latinx	13	57
Black/African American	11	48
Bicultural	7	30
LGBTQ+	5	22
Topic studied		
Obesity/Nutrition	8	35
Sexual and Reproductive Health	4	17
Mental Health	4	17
Healthcare Communication/Quality	3	13
Social Determinants in Health	2	9
Asthma	1	4
T1 Diabetes	1	4
Age		
Parent	11	48
Child	10	43
Adolescent	9	39
Community type		
Urban	13	57
Suburban	6	26
Rural	6	26
Region		
Northeast US	9	39
Southeast US	6	26
Midwest US	6	26
West US	4	17
Southwest US	3	13
International	2	9
Non-Continental US	1	4
Funding		
Federal	23	100
Non-Federal	8	35

We also compiled demographic information of participants included in this systematic review as provided in the original publications (Table 2). A total of 5,482 participants were reported across all 23 publications. Of studies reporting age, the average study participant age was 11.9 years old (range: 0.55–19.8 years) for the child and adolescent population and 35.5 years old for the parental population, with 55% of subjects being female and 45% male. As these publications were enriched to recruit and retain underrepresented pediatric populations, 2,676 (49%) were White while 2,488 (45%) were underrepresented populations. Black African American children accounted for 18% of reported study participants, Asian children accounted for 6%, and less than 2% for either Native American or Pacific Islander, while 18% of subjects reported were Hispanic/Latinx children.

**Table 2:** Demographics. Compiled demographics information for the populations reported in the 23 studies included in this systematic review.

Participant demographics (*N* = 5,482)	
Sex, *n* (%)	
Female	3,013 (55)
Male	2,455 (45)
Age, years, mean (min-max)	
Children	7.9 (0.55–13)
Adolescent	15.9 (13–19.8)
Parents	33.5 (32–35)
Age group, *n* (%)	
Children	2,387 (44)
Adolescent	1,446 (26)
Parents	1,649 (30)
Race, *n* (%)	
White	2,676 (49)
Black/African American	1,012 (18)
Multiracial	323 (6)
Asian	319 (6)
Pacific Islander	88 (2)
Native American	35 (1)
Other	711 (13)
Not reported	270 (5)
Ethnicity, *n* (%)	
Not-Hispanic	4,341 (79)
Hispanic	989 (18)
Not reported	125 (2)

### Pediatric recruitment strategies

**Figure 1:**
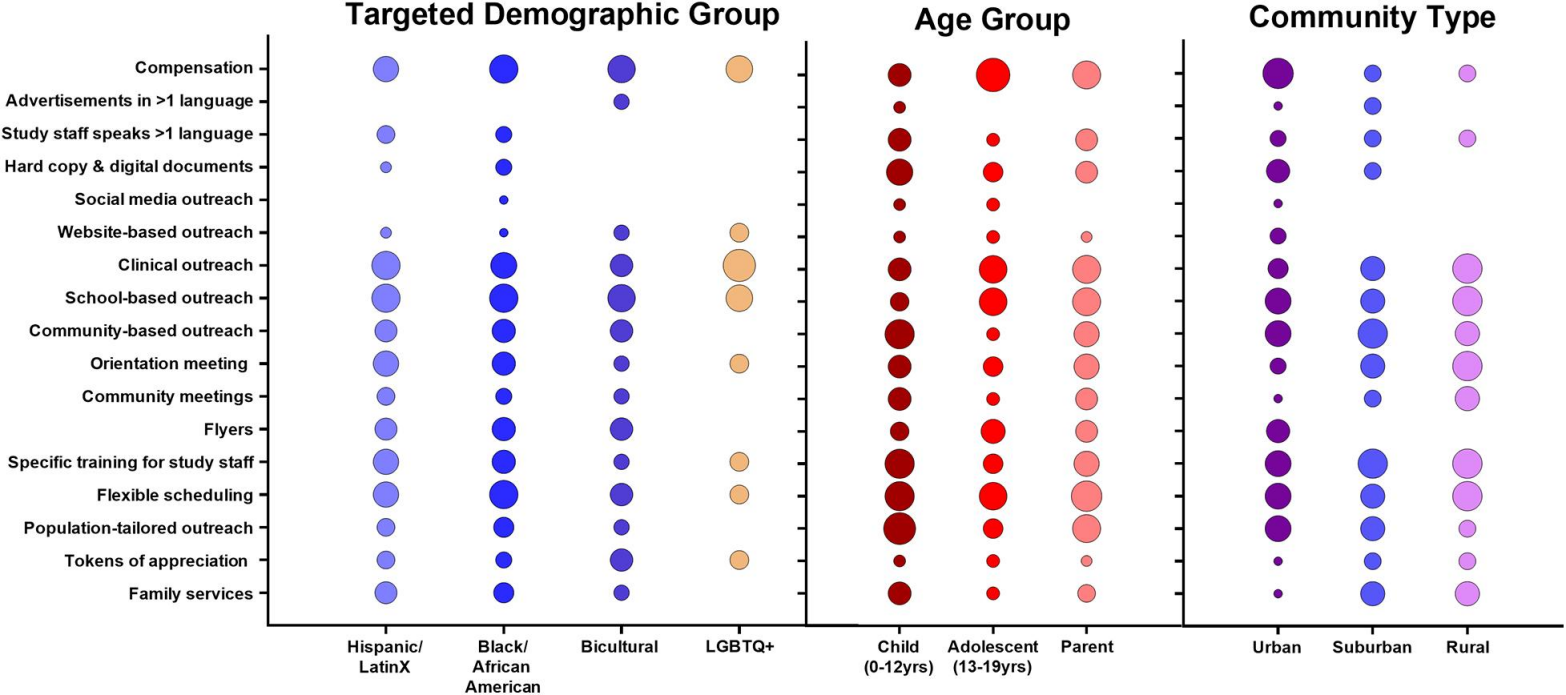
Frequency distribution of recruitment strategies stratified by demographic group, age cohort, and community location. Circle size represents relative frequency of use (standardized scale).

To assess for the most effective recruitment strategies, we evaluated studies' recruitment methods based on their use in targeted demographic groups (Hispanic/Latinx; Black African American; Bicultural; and LGBTQ+), age groups (children age 0–12 years; adolescent age 13–19 years; parent/guardian) and community type (urban; suburban; rural) (Figure 1). We also assessed frequency of methods used to recruit subjects according to study location, design, topic and funding source (Supplementary Figure S2). Commonly used strategies included monetary compensation, print-based/digital study materials, clinical and school-based outreach, community-based outreach, and flexible scheduling. Most studies employed multiple strategies, limiting direct assessment of individual methods' success; however, consistent patterns in recruitment effectiveness across demographic groups, age categories and community settings emerged.

#### Effective strategies for recruiting different age groups

**Figure 2:**
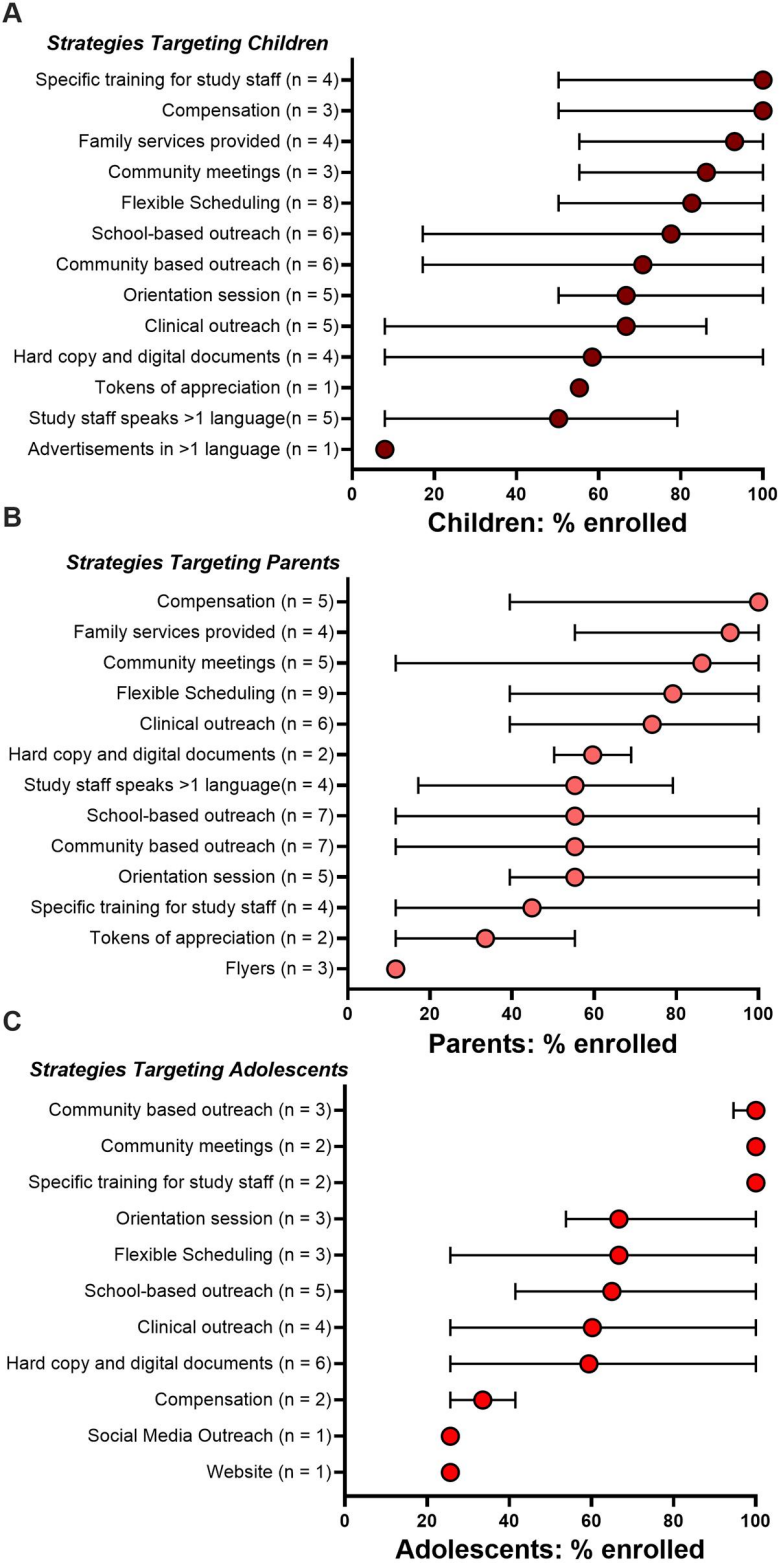
Efficacy of recruitment strategies used to recruit different age groups: **(A)** children <13 years, **(B)** parents, **(C)** adolescents. Strategies for each study and resultant percent of enrolled subjects were compiled. Median percent (and 95% conﬁdence intervals) of enrolled populations across all studies using each specific method are shown. Studies may have enrolled more than one age group and used more than one recruitment strategy.

We sought to evaluate which recruitment studies were associated with successful enrollment of different age groups (Figure 2). We focused on three age cohorts: young children ages 0–12 years; adolescents; and parents/guardians (with pediatric-focused research question).

For children ages 0–12 years, studies that included monetary compensation, specific training for study staff, and family services were the top recruiting strategies (100%, 100%, 93% enrollment of children, respectively) (Figure 2A). Community-based strategies also performed well, achieving 80% and 66% enrollment of young children. Strategies that were effective in recruiting children were also effective in recruiting for parent-targeted studies, suggesting that a combination of community-based approaches and addressing day-to-day needs is effective in recruiting both children and parents/guardians (Figure 2B). Other efforts such as flexible scheduling also demonstrated high effectiveness, with a median enrollment of 83% of children and 80% of parents/guardians, reflecting the importance of strategies that offer an opportunity for balancing economic, professional, and familial dynamics in parents' lives.

Adolescents demonstrated a strong alignment with community-based strategies (Figure 2B), including community meetings (100% median adolescent enrollment). School-based outreach, flexible scheduling, and clinical outreach showed moderate success, with median enrollment around 65%. Social networking platforms and websites, each used by only one study, had limited affect on this population, recruiting only about 25%. This suggests that group encounters play an important role for this population, while current web-based technologies social media platforms have not yet been optimized to serve as effective approaches for adolescents.

#### Efficacy of strategies enrolling racially and ethnically underrepresented populations

**Figure 3:**
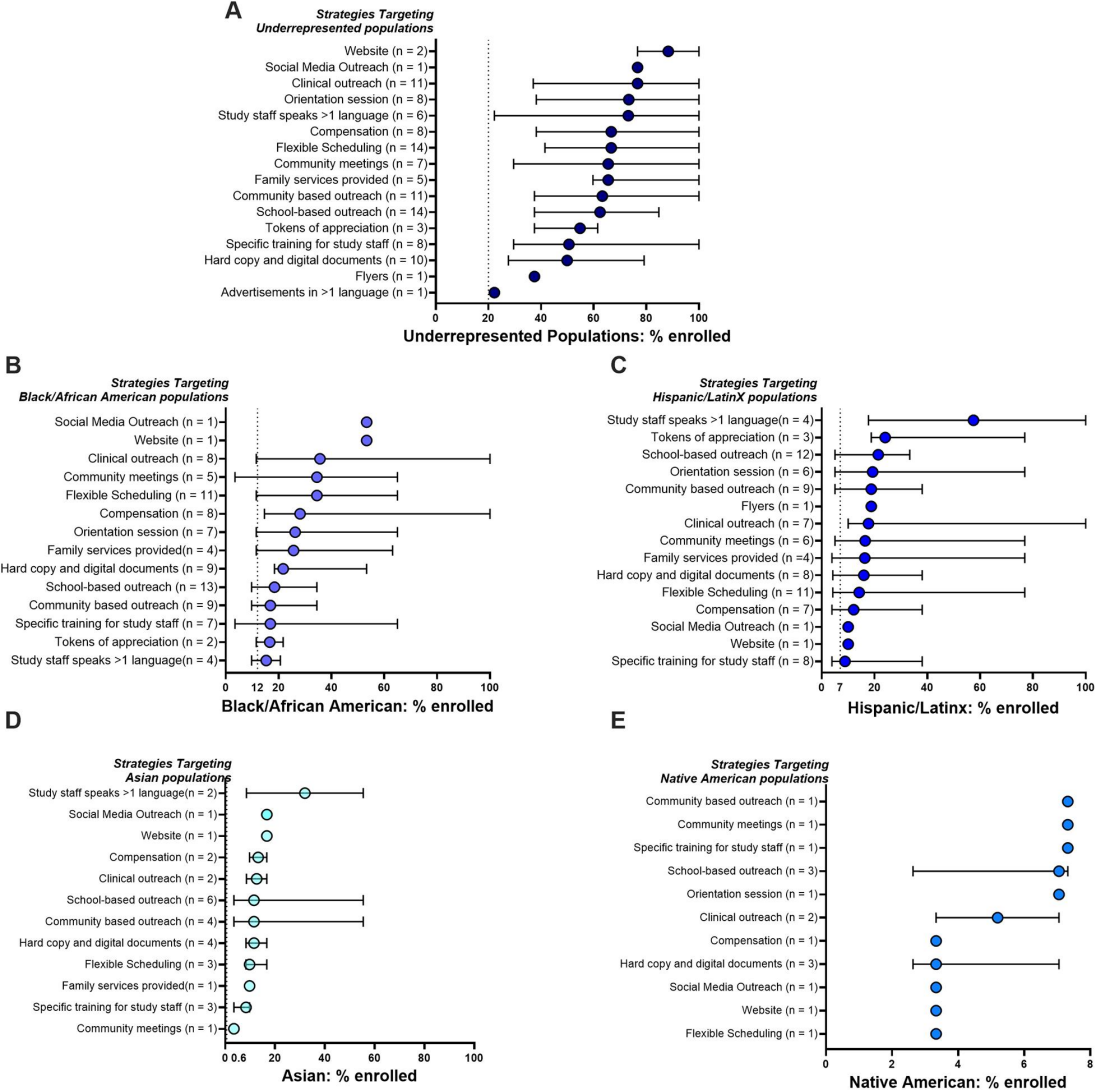
Efficacy of different recruitment strategies used in different populations: **(A)** combined underrepresented populations, **(B)** black African American, **(C)** Hispanic/Latinx, **(D)** Asian and **(E)** native American populations. Strategies for each study and resultant percent of enrolled subjects were compiled. Median percent (and 95% conﬁdence intervals) of enrolled populations across all studies using each specific method are shown. Studies may have used more than one recruitment tactic. The dotted line represents published national averages of underrepresented populations participating in pediatric clinical trials (40).

Our primary goal was to assess which recruitment strategies were associated with higher rates of recruiting underrepresented populations (Black African American, Hispanic/Latinx, Natives, Asian). Importantly, all studies included here reported recruitment of underrepresented populations as a priority, and recruitment efforts surpassed the 20% national average for underrepresented inclusion (40) [Figure 3A; dotted line represents national average (40)]. Social media and websites were highly effective, achieving 80%–90% recruitment of underrepresented groups, although used in only 1–2 studies each. Community-based strategies such as orientation sessions, community outreach, or community meetings recruited 65%–75% underrepresented populations when used alone or with other recruitment strategies. School-based outreach was frequently used (*n* = 14) and effective (62% underrepresented recruitment). Study specific methods, such as multilingual study staff or clinical outreach-trained staff recruited 73% and 76% underrepresented populations, respectively. Tokens of appreciation (such as thank you letters, bags) and monetary compensation were commonly used but varied widely in reported effectiveness, while multilingual advertisements were the least effective, barely surpassing current national underrepresented population benchmarks.

Recognizing cultural preferences and the variability of community interactions, we explored which strategies worked best within specific demographic groups (Figure 3A); the most effective strategies are highlighted in Table 3. For Black African American participants, effective strategies included social media and websites (53% Black African American participants each), clinical outreach (36%), community meetings (35%), flexible scheduling (34%), monetary compensation (28%), and family services (26%). Tokens of appreciation (16%) and multilingual advertisements (15%) were modestly effective (Figure 3B).

**Table 3:** Most effective recruitment strategies organized by demographic groups. Number of studies employing each method are listed, as are mean percent enrolled within each race/ethnic category.

Ethnicity/race	Recruitment strategy	Studies employing specified strategy	% enrolled
Hispanic/LatinX	Multilingual study staff	4	57
	Tokens of appreciation	3	24
	School-based outreach	12	21
	Orientation session	6	19
	Community-based outreach	9	19
	Flyers	2	19
Black African American	Social Media Outreach	1	53
	Website	1	53
	Clinical outreach	8	36
	Community meetings	5	34
	Flexible scheduling	12	33
	Compensation	9	31
Asian	Multilingual study staff	1	32
	Social Media Outreach	1	17
	Website	1	17
	Compensation	2	13
	Clinical outreach	2	13
	School-based outreach	6	12
Native American	Community-based outreach	1	7
	Community meetings	1	7
	Specific training for study staff	1	7
	School-based outreach	3	7
	Orientation session	1	7
	Clinical outreach	1	5

Strategies that were successful in recruiting Hispanic/Latinx subjects were distinct from those prioritizing recruitment of Black populations. Having Spanish-speaking staff was most effective (57% Hispanic/Latinx enrollment), while tokens of appreciation (24%), school-based outreach (21%), orientation sessions (19%), community-based outreach (19%), and monetary compensation (17%) had moderate success (Figure 3C).

Nationally, only 0.6% of study participants included in clinical trials identify as Asian (40), highlighting the marked underrepresentation of the numerous subgroups reporting the Asian racial designation (41). All studies analyzed in this review with Asian participants exceeded this rate (Figure 3D), suggesting focused efforts can improve Asian representation. Multilingual staff was most effective (31% Asian enrollment), with social media/websites (17%) slightly outperforming monetary compensation (13%), clinical outreach (12%), school-based outreach (12%), and community-based outreach (12%).

Native Americans are deeply underrepresented in pediatric clinical research (40). This review, too, found few studies including Native Americans. Community-based strategies (7% Native American enrollment), community meetings (7%), targeted staff training (7%), and clinical outreach (6%) were most effective, while school-based outreach varied in efficacy, and websites/social media had limited effect (3%) (Figure 3E).

#### Recruitment strategies for different community types

**Figure 4:**
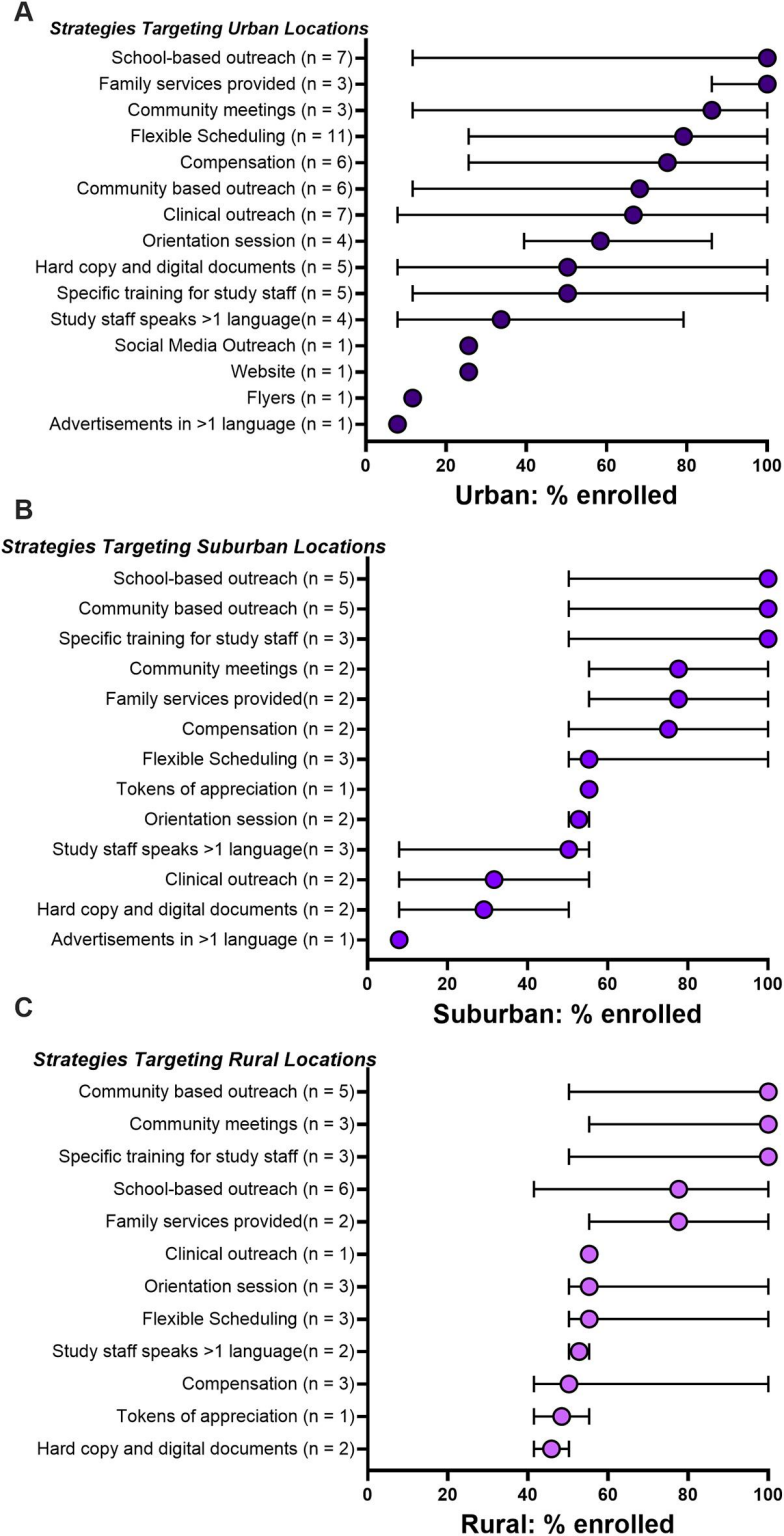
Efficacy of recruitment strategies used to recruit from different community types: **(A)** urban, **(B)** suburban, **(C)** rural. Strategies for each study and resultant percent of enrolled subjects were compiled. Median percent (and 95% conﬁdence intervals) of enrolled populations across all studies using each specific method are shown. Studies may have enrolled in more than one community type and used more than one recruitment strategy.

When seeking to enroll a diverse population, location, population density, and community resources are integral. Therefore, we assessed which recruitment strategies were most effective according to location area (urban, suburban, and rural) (Figure 4). We found that school-based outreach and family services were most effective at recruiting individuals from urban populations (100% urban enrollment each), followed closely by community meetings (86%), flexible scheduling (79%), and monetary compensation (75%) (Figure 4A). Studies involving suburban locations showed comparable results, with school-based outreach and community-based outreach achieving 100% suburban participant enrollment (Figure 4B). In rural locations (Figure 4C), community-based strategies such as community meetings and outreach were the most successful (100% median rural enrollment), followed by school-based outreach and family services (78% median enrollment). Monetary compensation and tokens of appreciation were less effective in rural populations.

### Pediatric retention strategies

#### Effective strategies for retaining different age groups

For both children and parents/guardians, tokens of appreciation were the most effective retention strategy, achieving a 100% retention rate in one study. Follow-up reminders also played a key role (84% retention across four studies), ensuring consistent communication. Family services (97%), receptiveness to feedback (97%), and flexible scheduling (86%) effectively retained adults and children (75% for each strategy). Monetary compensation was more effective retention strategy for parents (98% across four studies) than children (68% in one study). Among adolescents, flexible scheduling (94%), monetary compensation (90%), and follow-up reminders (90%) were the most effective retention strategies, highlighting the importance of incentives and regular reminders to maintain engagement (Figure 7A).

#### Strategic interventions for retaining racially and ethnically underrepresented populations

**Figure 5:**
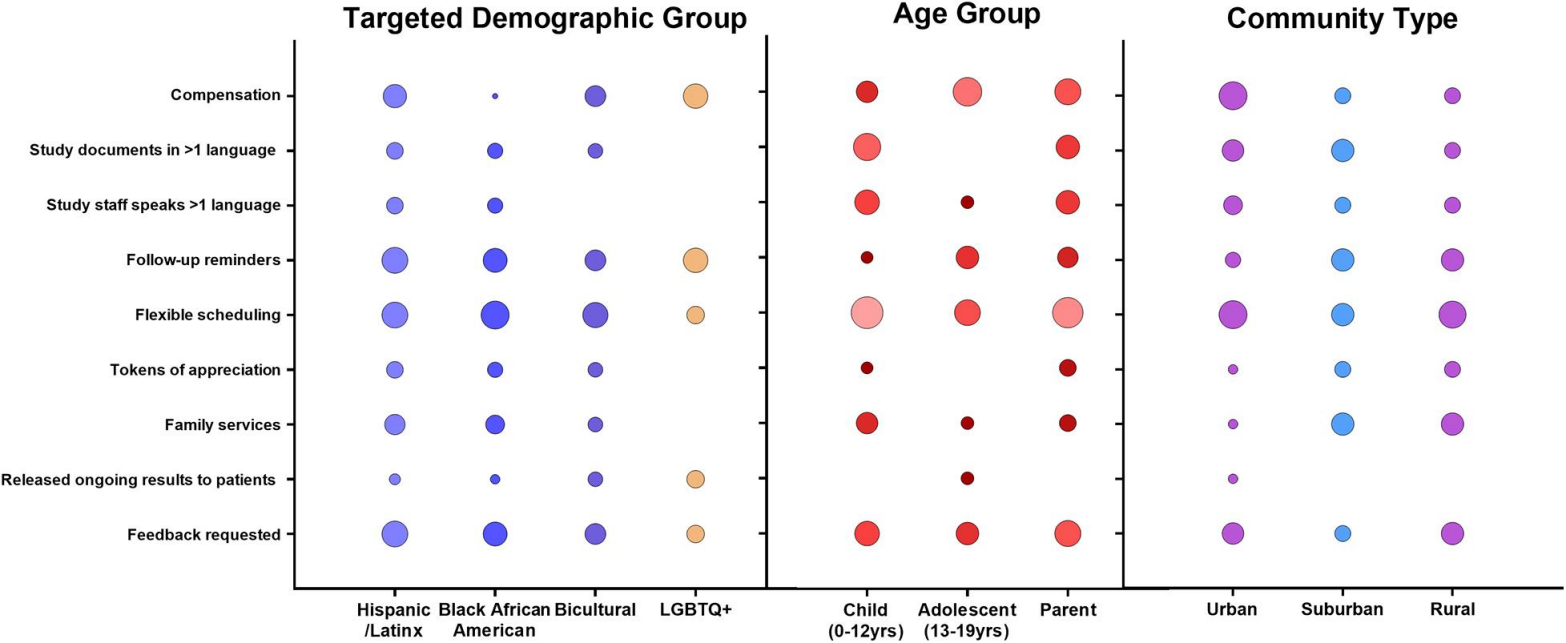
Frequency distribution of retention strategies across different demographics, ages and locations. Circle sizes have been standardized, ranging from 6% to 70% to represent the relative frequency of each recruitment strategy across categories.

While recruitment strategies gain interest of participants in a study, retention strategies focus on maintaining study engagement and require different strategies. Frequency of different retention strategies used by race/ethnicity, age, and community type are shown in Figure 5; Frequency of retention strategies used by study geographic location, design, topic and funding source are shown in Supplementary Figure S2. Fewer studies reported retention strategies and detailed demographics of individuals lost to follow-up. However, inclusion of demographic tables on both enrolled and retained subjects are important to understanding participation in clinical trials.

For underrepresented populations, multilingual study staff led to a 79% retention rate, showing the importance of language support for communication and trust. Monetary compensation for ongoing study participation was also effective (77% enrollment). Receptiveness to feedback (65%), family services (63%), and flexible scheduling (53%) and follow-up reminders were modestly effective (52%).

**Figure 6:**
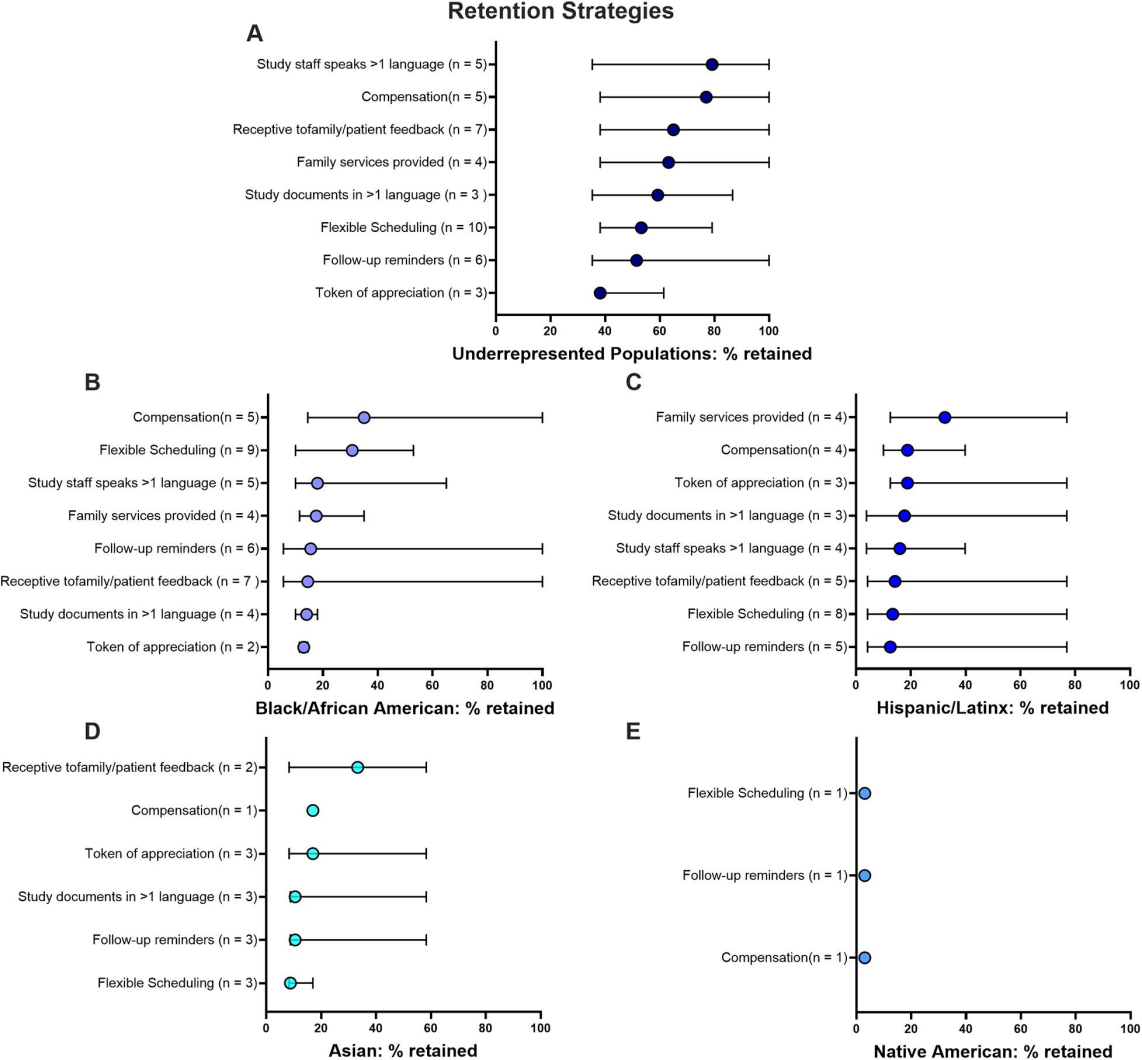
Efficacy of different retention strategies used in different populations: **(A)** combined underrepresented populations, **(B)** black African American, **(C)** Hispanic/Latinx, **(D)** Asian and **(E)** native American populations. Median percent (and 95% conﬁdence intervals) of retained populations across all studies using each specific method are shown. Studies may have used more than one recruitment tactic.

For Black African American populations, monetary compensation (35%) and flexible scheduling (30%) were most successful at retaining individuals. Among Hispanic/Latinx populations, family services were the most successful retention strategy (32%). In Asian populations, receptiveness to feedback was as effective as in the other groups (33%), indicating the importance of adapting to participant input. Lastly, among Native Americans, flexible scheduling, follow-up reminders, and monetary compensation all showed very low retention rates (3%), pointing to the need for more culturally tailored approaches to effectively engage this group (Figure 6).

#### Retention strategies for different community types

**Figure 7:**
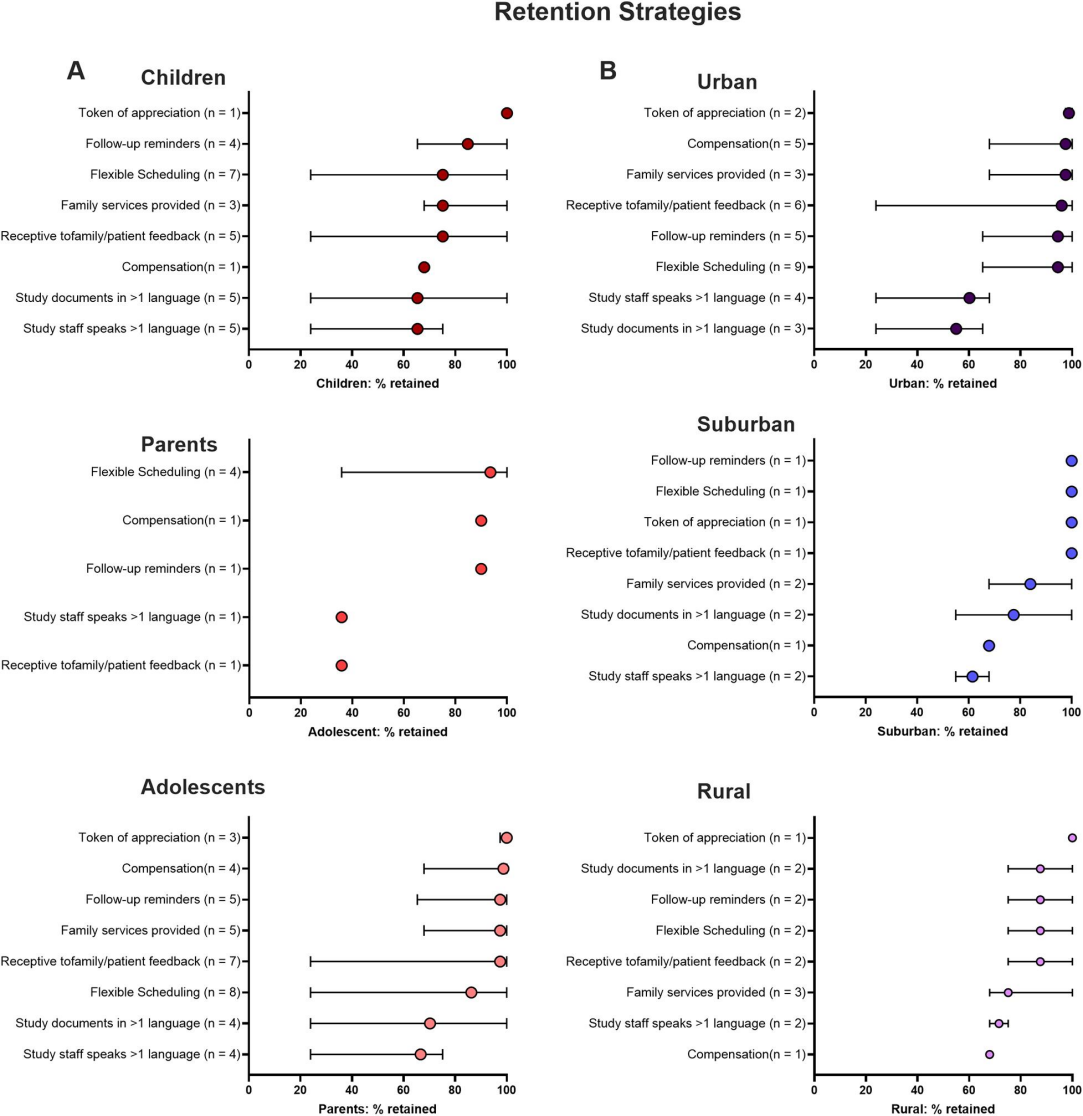
Efficacy of retention strategies used with different age groups: **(A)** children <13 years, parents, adolescents, and different locations: **(B)** urban, suburban, rural. Median percent (and 95% conﬁdence intervals) of retained subjects across all studies using each specific method are shown. Studies may have enrolled more than one age group/location and used more than one retention strategy.

Community location also revealed distinct retention needs (Figure 7B). In urban settings, tokens of appreciation, monetary compensation, family services, receptiveness to feedback, follow-up reminders, and flexible scheduling all performed well at retaining individuals, with greater than 95% retention across multiple studies. Many of the strategies were also successful in suburban areas, although family services and monetary compensation were less effective (84%, 68% retention, respectively) compared to urban areas. In rural areas, family services were similarly less effective (75%), while tokens of appreciation (100%) were most effective. Study documents in multiple languages, follow-up reminders, flexible scheduling, and receptiveness to feedback were more effective (88%), emphasizing the importance of communication and flexibility.

## Discussion

Here, we report a systematic review of publications aimed at enrolling diverse cohorts of children in pediatric research with the goal of highlighting successful strategies for recruiting and retaining underrepresented populations in pediatric clinical studies. Throughout this analysis, consistent patterns emerged regarding the effectiveness of certain strategies, particularly those that promote direct interaction and trust between researchers and participants. Additionally, a clear distinction was observed between strategies aimed at recruiting vs. retaining participants, underscoring the importance of tailoring interventions to specific goals.

A key observation was the variation in strategies employed, ranging from those that prioritized study design and preparation to those that emphasized direct community engagement. Strategies such as having a multilingual staff, providing documents in multiple languages, offering flexible scheduling or maintaining a social media presence required significant planning, funding, and effort. These approaches aimed to make the study more accessible to participants, focusing on logistical accessibility rather than fostering direct engagement. On the other hand, approaches like community meetings, school-based outreach, clinical outreach, family services, and monetary compensation often addressed the challenge of generating trust and long-term commitment. By incorporating support services such as transportation, childcare and food access, these approaches eased participation for families. Embedding these support services within the community fosters trust, belonging, and representation, which are essential for research participation.

Community-based strategies were particularly central to successful recruitment of racially and ethnically diverse populations. These strategies enable personal and direct interaction where participants can voice their concerns, meaningfully participate in conversations, and feel heard. The preference for community-based strategies suggests that participants value social connections and trust-building. Community strategies foster a sense of belonging and mutual respect (42), which enhance participant motivation. These kinds of approaches do not merely facilitate the enrollment logistics, but they actively restructure the relational dynamic between researchers and communities, transforming research participation from a transactional interaction into a trust based partnership. By valuing and respecting participants' time, culture, and experiences, a dynamic of reciprocal trust is created. This trust can be a decisive factor in overcoming initial resistance that some individuals may have toward participating in research studies. Concerns related to data privacy, for example, may be a barrier to participation (12), especially as they relate to children; this can be addressed through community efforts to foster both recruitment and long-term engagement in research.

We also identified key differences in strategies for recruiting different age groups. We found many similarities between successful strategies used for children and parents/guardians, as studies involving children depend on parental participation. Integrating research efforts within schools was successful for recruitment, as schools provide a trusted environment and consistent points of contact between researchers and families. Adolescents, however, preferred activities including peers, such as community meetings and orientation sessions, where they can receive support from friends and family while also exhibiting autonomy.

In contrast to recruitment, successful retention relied on sustained support strategies, focusing on maintaining ongoing engagement throughout the study. The goal of retention is not to convince participants to join but to facilitate their continued participation by reducing barriers that may arise over time, allowing balance in participants’ involvement with their daily responsibilities. This comprehensive support system includes practical assistance such as reminders, financial incentives, transportation, childcare, and flexible scheduling allowing participants to stay engaged without the study imposing additional burdens on their everyday lives. These sustained support strategies reduce the burden of participation and foster a collaborative experience in which participants feel their contributions are valued. This balance is crucial to retaining a diverse and engaged study population that feels genuinely supported and respected.

Monetary compensation was one of the most common strategies. While remuneration for individuals' time and effort is important, our findings suggest that compensation played a more consistent role in retention rather than initial recruitment. This pattern may reflect underlying behavioral reinforcement principles where sustained engagement is supported by tangible incentives (43). These findings suggest that financial incentives alone may be insufficient to overcome initial structural or relational barriers, but may help sustain engagement once participation has begun. This indicates that while people may be initially motivated by community connection and a sense of belonging, financial incentives become key to their long-term participation.

The concept of “underrepresented” populations is highly region-dependent and may take different forms in international research settings. Outside the U.S., underrepresentation may be driven less by race or ethnicity alone and more by factors such as rurality, linguistic barriers, migration status, socioeconomic marginalization, historical exclusion, or political instability. Extensive work in global health fields such as HIV and substances use research has demonstrated that similar structural barriers (such as mistrust on research institutions, stigma and limited access to healthcare) shape participation across diverse contexts (11, 44, 45). Importantly, many of the strategies highlighted in this review, including community engagement, trust building and provision of practical supports, have been successfully adapted in international contexts despite the differences in how underrepresentation is defined. Recognizing the contextual nature of the underrepresentation underscores the broader applicability of these principles and support its relevance beyond pediatric research based in U.S.

Although this systematic review highlights successful recruitment and retention strategies for enrolling diverse pediatric populations, we did encounter limitations. First, we relied on reported data for enrolled individuals. We did not have data on recruited but not enrolled individuals, therefore we cannot compare the total number approached within specific communities to ensure adequate enrollment numbers. More detailed descriptions of recruited, rather than only enrolled, populations would assist in generating more tailored and effective recruitment strategies. Diversity also could have been influenced by an individual study's recruitment targets. For instance, studies aiming to exclusively enroll Black participants, for example, will achieve higher recruitment for that racial group compared to studies aiming for broader diversity across multiple groups. Additionally, many studies employed multiple recruitment strategies, so it is unclear which individual strategies confer highest recruitment rates. Moreover, current classifications do not account for individuals identifying with multiple ethnic or racial groups (36), which may evolve across generations, highlighting the need for more inclusive classification systems. Finally, many studies lacked detailed demographics on those lost to follow-up, complicating the assessment of whether retention strategies effectively retained these populations, thus limiting our findings and warranting further exploration in future studies.

## Conclusion

In summary, this systematic review highlights distinct recruitment and retention strategies to broadly recruit for pediatric clinical and translational research. Community-based strategies were shown to serve as a highly successful recruitment tool, likely by establishing an essential pillar for building trust and maintaining long-term participation. Monetary compensation and other logistical incentives, such as follow-up reminders and family services, played a more substantial role in retention. Learning from the successes of prior pediatric studies will undoubtedly maximize recruitment of underrepresented populations in pediatric research, thereby ensuring adequate representation of children from various backgrounds that will benefit generations to come.

## Data Availability

The original contributions presented in the study are included in the article/Supplementary Material, further inquiries can be directed to the corresponding author.

## Supplementary material

Supplementary Figure S1 PRISMA flow diagram for the identification of studies included in this systematic review.

Supplementary Figure S2 Frequency distribution of recruitment and retention strategies across different study types, study topics, location and funding source. Circle sizes have been standardized, ranging from 5% to 100% to represent the relative frequency of each recruitment strategy across categories.
