# Supplementary figures and images for: Strategic recruitment and retention for pediatric research: a systematic review and meta-analysis
Source: Front Pediatr. 2026 Apr 10;14:1786388. doi: 10.3389/fped.2026.1786388 (PMC13106582; doi:10.3389/fped.2026.1786388)

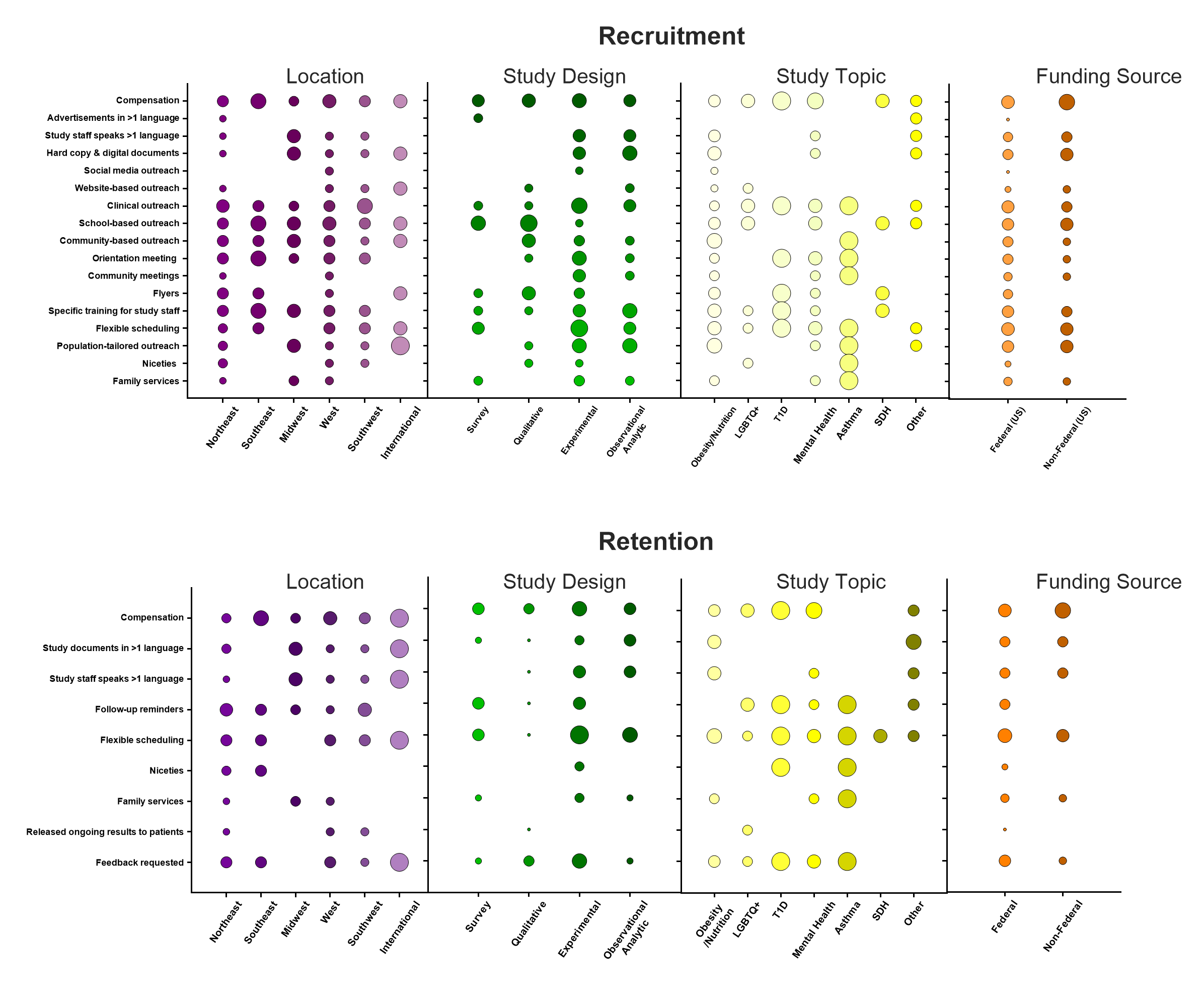

Supplement: Supplementary Figure S1 — PRISMA flow diagram for the identification of studies included in this systematic review. [file Image1.tif]
