# Supplementary material for: Strategic recruitment and retention for pediatric research: a systematic review and meta-analysis
Source: Front Pediatr. 2026 Apr 10;14:1786388. doi: 10.3389/fped.2026.1786388 (PMC13106582; doi:10.3389/fped.2026.1786388)
Supplement: Supplementary Figure S2 — Frequency distribution of recruitment and retention strategies across different study types, study topics, location and funding source. Circle sizes have been standardized, ranging from 5% to 100% to represent the relative frequency of each recruitment strategy across categories. [file Datasheet1.pdf]

Strategy	Phase (Recruitment/ Retention/Both)	Operational Definition	Representative Examples
Compensation	Both	Financial remuneration provided for enrollment or continued participation	Gift cards at baseline and/or follow-up visits
Tokens of appreciation	Both	Small non-monetary tokens of appreciation	Snacks, small gifts, appreciation items
Study staff speaks >1 language	Both	Bilingual or multilingual staff facilitating communication and engagement	Spanish-speaking research coordinators
Flexible Scheduling	Both	Accommodation of participant availability to reduce participation burden	Evening or weekend appointments
Advertisements in >1 language	Recruitment	Study advertisements available in multiple languages	Spanish-language flyers
Website	Recruitment	Study-specific websites to disseminate study information and enroll participants	Study webpage with enrollment link
Social Media Outreach	Recruitment	Recruitment via social media platforms	Facebook/Instagram study advertisements
Clinical outreach	Recruitment	Recruitment during clinical encounters in general pediatric settings	Enrollment during well-child visits
Orientation session	Recruitment	Informational sessions provided prior to enrollment to explain study procedures	Pre-enrollment informational meetings
Specific training for study staff	Recruitment	Staff training in cultural competence or community engagement	Cultural responsiveness workshops
Hard copy and digital documents	Recruitment	Distribution of printed and electronic study materials	Brochures, flyers, email PDFs
Flyers	Recruitment	Printed advertisements distributed in community locations	Informational flyers
Community meetings	Recruitment	In-person engagement sessions with community members or leaders	Town-hall style information sessions
Community-based outreach	Recruitment	Recruitment conducted in trusted community settings	Outreach at community centers
School-based outreach	Recruitment	Recruitment conducted within school systems	Classroom presentations
Study documents in >1 language	Retention	Consent forms or study materials translated for ongoing participation	Multilingual follow-up documents
Follow-up reminders	Retention	Structured communication to encourage continued participation	SMS reminders; reminder calls; emails
Receptive to family/patient feedback	Retention	Incorporation of participant feedback to improve study experience	Adjustments based on parent suggestions
Family services provided	Retention	Support services aimed at reducing logistical burden	Transportation vouchers
