# Supplementary material for: Strategic recruitment and retention for pediatric research: a systematic review and meta-analysis
Source: Front Pediatr. 2026 Apr 10;14:1786388. doi: 10.3389/fped.2026.1786388 (PMC13106582; doi:10.3389/fped.2026.1786388)
Supplement: Supplementary file 3 [file Datasheet2.pdf]

Author	PMID	Year of publication	Title	Health topic studied	Recruitment Strategy	Retention Strategy
Diane Berry et al.	23294390	2013	Recruitment and retention strategies for a community-based weight management study for multi-ethnic elementary school children and their parents	Obesity/ Nutrition	• Study staff speaks >1 language<br>• Provider/clinical outreach<br>• Orientation session/informational meeting<br>• Community meetings/panels<br>• Study-specific training/education for study staff<br>• Flexible scheduling<br>• Family services provided	• Study documents in >1 language<br>• Study staff speaks >1 language<br>• Flexible scheduling<br>• Family services provided<br>• Receptive to community patient feedback
Carrie Nieman et al.	23853050	2013	Whose experience is measured? A pilot study of patient satisfaction demographics in pediatric otolaryngology	Other	• Advertisements in >1 language	• Study documents in >1 language
Nicole Bush et al.	27842184	2017	Socioeconomic Disparities in Childhood Obesity Risk: Association With an Oxytocin Receptor Polymorphism	Obesity/ Nutrition	• Compensation<br>• School-based outreach<br>• Flexible scheduling	• Compensation<br>• Flexible scheduling
Rachel M. Burke et al.	29530004	2018	Effect of infant feeding practices on iron status in a cohort study of Bolivian infants	Obesity/ Nutrition	• Compensation<br>• Study materials available hard copy and/or digitally<br>• Website-based outreach<br>• Flexible scheduling<br>• Outreach tailored to specific demographic group	• Compensation<br>• Flexible scheduling
Sagatov et al.	30209890	2018	Recruitment outcomes, challenges and lessons learned: the Healthy Communities Study	Obesity/ Nutrition	• School-based outreach<br>• Community center-based outreach<br>• Orientation session/informational meeting<br>• Study-specific training/education for study staff	None
Miranda Pallan et al.	31293236	2019	Cultural adaptation of an existing children's weight management programme: the CHANGE intervention and feasibility RCT	Obesity/ Nutrition	• School-based outreach<br>• Community center-based outreach<br>• Printed flyers<br>• Outreach tailored to specific demographic group	• Study documents in >1 language<br>• Study staff speaks >1 language<br>• Flexible scheduling<br>• Receptive to community patient feedback
Sarah Marshall et al.	30786969	2019	Social Context of Sexual Minority Adolescents and Relationship to Alcohol Use	LGTBQ+	• Compensation<br>• School-based outreach	• Compensation
Malia Shimokawa et al.	32490382	2020	The NEW Keiki Program Reduces BMI z-scores Among Overweight and Obese Children and BMI Among Their Adult Caregivers in Hawai'i	Obesity/ Nutrition	• Compensation<br>• Study staff speaks >1 language<br>• Study materials available hard copy and/or digitally<br>• Provider/clinical outreach<br>• Community meetings/panels<br>• Study-specific training/education for study staff<br>• Flexible scheduling<br>• Outreach tailored to specific demographic group	• Compensation<br>• Study documents in >1 language<br>• Study staff speaks >1 language<br>• Flexible scheduling<br>• Receptive to community patient feedback
Nicole Fearnbach et al.	32335525	2020	A Pilot Study of Cardiorespiratory Fitness, Adiposity, and Cardiometabolic Health in Youth With Overweight and Obesity	Obesity/ Nutrition	• Provider/clinical outreach<br>• Orientation session/informational meeting	None
Brittany Allen et al.	32105347	2020	At the Margins: Comparing School Experiences of Nonbinary and Binary-Identified Transgender Youth	LGTBQ+	• Compensation<br>• Study materials available hard copy and/or digitally<br>• Social media outreach<br>• Community center-based outreach<br>• Printed flyers<br>• Flexible scheduling<br>• Outreach tailored to specific demographic group	• Compensation<br>• Flexible scheduling
Ushma Upadhyay et al.	32539726	2020	Using online technologies to improve diversity and inclusion in cognitive interviews with young people	LGTBQ+	• Compensation<br>• Study materials available hard copy and/or digitally<br>• Website-based outreach<br>• Flexible scheduling<br>• Outreach tailored to specific demographic group	• Compensation<br>• Flexible scheduling
Sheree Toth et al.	32696103	2020	The Moderating Role of Child Maltreatment in Treatment Efficacy for Adolescent Depression	Mental Health	• Compensation<br>• Study staff speaks >1 language<br>• Study materials available hard copy and/or digitally<br>• Provider/clinical outreach<br>• Orientation session/informational meeting<br>• Community meetings/panels<br>• Flexible scheduling<br>• Outreach tailored to specific demographic group<br>• Courteous outreach/niceties<br>• Family services provided	• Compensation<br>• Study staff speaks >1 language<br>• Flexible scheduling<br>• Receptive to community patient feedback
Caitlin Shneider et al.	34237457	2021	Recruiting and retaining parents in behavioral intervention trials: Strategies to consider	T1 Diabetes	• Compensation<br>• Provider/clinical outreach<br>• Orientation session/informational meeting<br>• Printed flyers<br>• Study-specific training/education for study staff<br>• Flexible scheduling	• Compensation<br>• Follow-up reminders<br>• Flexible scheduling (online visits, flexible hours, short duration)<br>• Niceties provided (thank you letters, etc.)<br>• Receptive to community patient feedback
Rose Wesche et al.	33260054	2021	Developing an inclusive Safe Dates program for sexual and gender minority adolescents: A pilot study	Mental Health	• Provider/clinical outreach<br>• Orientation session/informational meeting	None
G Alice Woolverton et al.	34351178	2021	"I just check 'other'": Evidence to support expanding the measurement inclusivity and equity of ethnicity/race and cultural identifications of U.S. adolescents	Social Determinants of Health	• Compensation<br>• School-based outreach<br>• Printed flyers	None
Santana Silver et al.	35624421	2022	Stakeholder engagement in developing a father-inclusive early life obesity prevention intervention: First Heroes	Obesity/ Nutrition	• Compensation<br>• Study staff speaks >1 language<br>• Study materials available hard copy and/or digitally<br>• Provider/clinical outreach<br>• Orientation session/informational meeting<br>• Community meetings/panels<br>• Study-specific training/education for study staff<br>• Flexible scheduling	Compensation
Julia O'Donoghue et al.	35995130	2022	Strategies to improve the recruitment and retention of underserved children and families in clinical trials: A case example of a school-supervised asthma therapy pilot	Asthma	• Provider/clinical outreach<br>• Community center-based outreach<br>• Orientation session/informational meeting<br>• Community meetings/panels<br>• Flexible scheduling<br>• Outreach tailored to specific demographic group<br>• Courteous outreach/niceties<br>• Family services provided	• Follow-up reminders<br>• Flexible scheduling (online visits, flexible hours, short duration)<br>• Niceties provided (thank you letters, etc.)<br>• Family services provided<br>• Receptive to community patient feedback
Melanie Killen et al.	35612354	2022	Testing the effectiveness of the Developing Inclusive Youth program: A multisite randomized control trial	Social Determinants of Health	• School-based outreach	None
Amy Damashek et al.	37122442	2022	Tailoring a child injury prevention program for low-income U.S. families.	Other	• Compensation<br>• School-based outreach<br>• Printed flyers<br>• Flexible scheduling<br>• Family services provided	• Compensation<br>• Follow-up reminders<br>• Flexible scheduling<br>• Family services provided<br>• Receptive to community patient feedback
Dalmacio Flores et al.	37062572	2023	Inclusive and Age-Appropriate Timing of Sexual Health Discussions at Home According to Gay, Bisexual, and Queer Adolescent Males	LGTBQ+	• School-based outreach<br>• Community center-based outreach<br>• Printed flyers<br>• Outreach tailored to specific demographic group	None
Vivian Villegas et al.	37639038	2023	Diversified caregiver input to upgrade the Young Children's Participation and Environment Measure for equitable pediatric re/habilitation practice	Mental Health	• Compensation<br>• Website-based outreach<br>• Provider/clinical outreach	• Compensation<br>• Follow-up reminders<br>• Gave patients ongoing results<br>• Receptive to community patient feedback
Arthur Andrews et al.	36862478	2023	Clinical diversity in a randomized trial that explicitly sought racial/ethnic diversity in its sample: Baseline comparisons in a treatment of youth substance use and posttraumatic stress	Mental Health	• Study staff speaks >1 language<br>• Study materials available hard copy and/or digitally<br>• Community center-based outreach<br>• Study-specific training/education for study staff<br>• Flexible scheduling<br>• Outreach tailored to specific demographic group<br>• Family services provided	• Study documents in >1 language<br>• Study staff speaks >1 language<br>• Family services provided

Chelsea Wynn et al.	37389945	2023	Examination of Text Message Plans and Baseline Usage of Families Enrolled in a Text Message Influenza Vaccine Reminder Trial: Survey Study	Other	• Study staff speaks >1 language<br>• Study materials available hard copy and/or digitally<br>• Provider/clinical outreach<br>• Outreach tailored to specific demographic group	• Study documents in >1 language<br>• Study staff speaks >1 language<br>• Follow-up reminders
