# Supplementary material for: Strategic recruitment and retention for pediatric research: a systematic review and meta-analysis
Source: Front Pediatr. 2026 Apr 10;14:1786388. doi: 10.3389/fped.2026.1786388 (PMC13106582; doi:10.3389/fped.2026.1786388)

PRISMA 2020 flow diagram for new systematic reviews which included searches of databases and registers only

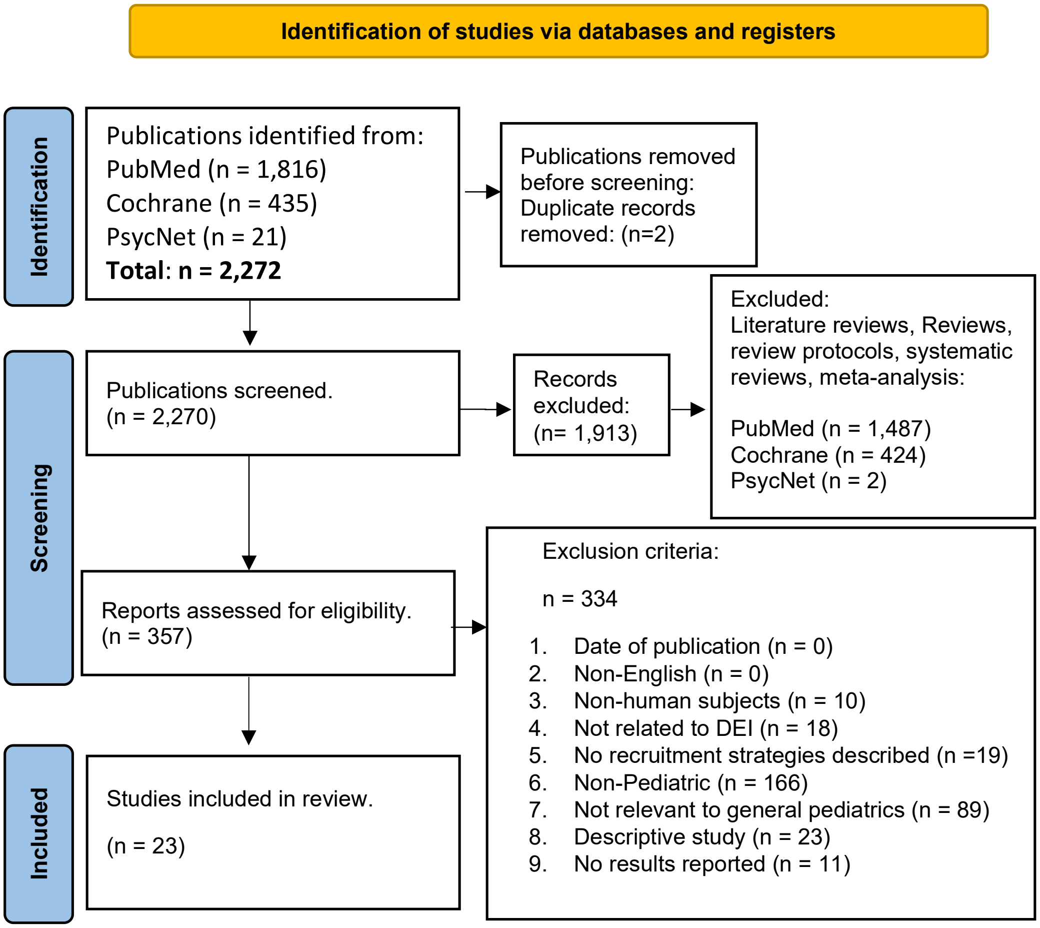

Supplement: Supplementary file 4 [file Datasheet3.pdf]
